# Fine mapping and replication of QTL in outbred chicken advanced intercross lines

**DOI:** 10.1186/1297-9686-43-3

**Published:** 2011-01-17

**Authors:** Francois Besnier, Per Wahlberg, Lars Rönnegård, Weronica Ek, Leif Andersson, Paul B Siegel, Orjan Carlborg

**Affiliations:** 1 Department of Animal Breeding and Genetics, Swedish University of Agricultural Sciences, Uppsala, Sweden; 2 Department of Medical Biochemistry and Microbiology, Uppsala University, Uppsala, Sweden; 3 Statistics Unit, Dalarna University, Borlänge, Sweden; 4 Virginia Polytechnic Institute and State University, Department of Animal and Poultry Sciences, Blacksburg, VA 24061-0306, USA; 5 Linnaeus Centre for Bioinformatics, Uppsala University, SE-75124 Uppsala, Sweden

## Abstract

**Background:**

Linkage mapping is used to identify genomic regions affecting the expression of complex traits. However, when experimental crosses such as F_2 _populations or backcrosses are used to map regions containing a Quantitative Trait Locus (QTL), the size of the regions identified remains quite large, i.e. 10 or more Mb. Thus, other experimental strategies are needed to refine the QTL locations. Advanced Intercross Lines (AIL) are produced by repeated intercrossing of F_2 _animals and successive generations, which decrease linkage disequilibrium in a controlled manner. Although this approach is seen as promising, both to replicate QTL analyses and fine-map QTL, only a few AIL datasets, all originating from inbred founders, have been reported in the literature.

**Methods:**

We have produced a nine-generation AIL pedigree (*n *= 1529) from two outbred chicken lines divergently selected for body weight at eight weeks of age. All animals were weighed at eight weeks of age and genotyped for SNP located in nine genomic regions where significant or suggestive QTL had previously been detected in the F_2 _population. In parallel, we have developed a novel strategy to analyse the data that uses both genotype and pedigree information of all AIL individuals to replicate the detection of and fine-map QTL affecting juvenile body weight.

**Results:**

Five of the nine QTL detected with the original F_2 _population were confirmed and fine-mapped with the AIL, while for the remaining four, only suggestive evidence of their existence was obtained. All original QTL were confirmed as a single locus, except for one, which split into two linked QTL.

**Conclusions:**

Our results indicate that many of the QTL, which are genome-wide significant or suggestive in the analyses of large intercross populations, are true effects that can be replicated and fine-mapped using AIL. Key factors for success are the use of large populations and powerful statistical tools. Moreover, we believe that the statistical methods we have developed to efficiently study outbred AIL populations will increase the number of organisms for which in-depth complex traits can be analyzed.

## Background

In domestic animal populations, F_2 _crosses between divergently selected outbred lines are commonly used to map QTL [1-3]. However, only one generation of recombination occurs, in an F_2 _pedigree (gametes of the F_1 _generation) and linkage disequilibrium (LD) can be strong along the chromosomes. This long-range LD can be used to detect associations between QTL and markers even at a low marker density, e.g. one marker per 10 or 20 centiMorgans (cM) [1,4]. However, because of the extensive LD, using an F_2 _design results in large confidence intervals for QTL locations [5] that potentially contain hundreds of genes.

To map QTL with a higher resolution, it is necessary to adopt a fine-mapping strategy that would ideally produce a QTL peak covering a chromosome region small enough to contain only a few genes. Such a strategy would facilitate identification of candidate mutations. Precision of fine-mapping relies on the use of dense SNP marker maps that provide genotypic information at cM or sub-cM intervals. However, using a high or medium high marker density in an F_2 _population provides only a moderate improvement in the resolution because the population has undergone only one generation of recombination [5]. In such a population, most of the marker alleles are inherited together and share the same genetic information i.e. they are Identical By Descent (IBD) within the same haplotype block [6]. Reducing the extensive linkage disequilibrium present in an F_2 _population requires breeding additional filial generations by repeated intercrossing, i.e. using F_2 _individuals to generate an F_3 _generation and so on, to form an Advanced Intercross Line (AIL) pedigree [6-8]. Each generation of breeding introduces new recombination events and thereby decreases LD between markers and QTL. Thus, an AIL makes it possible to re-analyze and fine-map QTL originally detected in the F_2 _generation.

Here, we developed new methods to fine-map QTL using data from an AIL produced from outbred lines. We used a nine-generation AIL pedigree produced from an intercross between two lines of chickens divergently selected for body weight at 56 days of age [9]. All animals in the pedigree were genotyped using SNP markers located at approximately 1 cM intervals in nine chromosomal regions where significant or suggestive QTL had previously been identified in an independent F_2 _intercross between the lines [10,11]. The nine regions were screened for QTL influencing body weight at 56 days of age (BW56), with the objective of replicating previous results and reducing the size of the confidence intervals containing the QTL.

## Methods

### Animals

To create the AIL used in this study, a large intercross pedigree was set up by crossing individuals from two divergent chicken lines, i.e. a High Weight Selected line (HWS) and a Low Weight Selected line (LWS), which were obtained as follows. A selection experiment initiated in 1957 was designed to select two chicken lines for high- and low-body weight from the same base population, which consisted of crosses between seven partly inbred lines of White Plymouth Rock chickens. Then, individuals with a high-body weight at 56 days of age (BW56) were selected as parents for the line HWS and chickens with a low BW56 were selected as parents for the line LWS [9].

The AIL was initiated with HWS and LWS individuals from generation 40 [10-12] for which sex-averaged mean body weights at the age of selection were1522 g (SE: ± 36 g) for HWS and 181 g (SE: ± 5 g) for LWS animals. The observed mean heterozygosity, H_0_, at all autosomal loci, after 40 generations, was 0.146 in line HWS and 0.156 in line LWS [13]. To create the AIL, 10 HWS males were mated with 22 LWS females and 8 LWS males were mated with 19 HWS females to produce 83 F_1_. The number of individuals produced in subsequent generations varied (Table 1); about 100 individuals for generations F_2_, F_4_, F_5_, F_6 _and F_7_; 405 individuals for generation F_8 _because F_8 _animals have accumulated the highest number of recombinations and are expected to provide the best resolution for QTL mapping; and 437 individuals for generation F_3_, which is a suitable size to detect QTL in regions where genetic polymorphism may have been lost by genetic drift during the last generations of the intercross. Breeding conditions differed for producing the F_8 _versus previous generations, i.e. fewer sires and younger dams were used to produce the F_8_, resulting in smaller egg size and ultimately smaller animals (Table 1). This was accounted for in our statistical model by correcting for a generation effect.

**Table 1:** Descriptive statistics for the AIL pedigree

Generation	Nb of animals	Nb of males contributing to the next generation	Average body weight at 56 days of age (g) ± SE
F0 HWS	29	10	1522 ± 36
F0 LWS	30	8	181 ± 5.4
F1	83	8	NA
F2	90	32	663.9 ± 18.6
F3	437	65	696.7 ± 8.1
F4	128	58	594.6 ± 11.9
F5	128	51	656.1 ± 14.0
F6	100	35	770.1 ± 16.9
F7	107	10	661.8 ± 17.2
F8	405	NA	373.6 ± 6.3
**Total**	**1529**	NA	**594.2 ± 5.7**

### DNA extraction, marker selection and genotyping

Nine chromosome regions (see Table 2; segment names follow the Jacobsson et al. [10] nomenclature) containing significant or suggestive QTL for body weight detected in the original F_2 _population [10,11] were chosen for this study. DNA was extracted from blood samples by AGOWA (Berlin, Germany). Fifteen individuals from each parental line were genotyped for approximately 13,000 genome-wide SNP markers, as described by Wahlberg et al. [14]. A subset of 304 segregating SNP was selected from the nine QTL regions to, in the best possible way, discriminate between alleles inherited from the HWS and LWS lines. For SNP markers that are bi-allelic, a marker that discriminates between alleles inherited from the HWS and LWS lines would have a high frequency (p) of one allele in the LWS line, and a high frequency (q) of the other allele in the HWS line. In the ideal case, p(HWS) = q(LWS) = 1 (i.e. fixation for alternative alleles in the two lines), the molecular signature of the markers is sufficient to determine the line of origin of any allele without uncertainty. As this situation rarely occurred for the markers available in the present study, markers were selected as follows: First, differences in marker allele-frequencies between the HWS and LWS lines were evaluated for all markers in the QTL regions. Then, markers were selected based on decreasing differences in marker allele frequencies between the lines, and on their positions at regular intervals on the chromosome segments. In the present study, only 33% of the markers were considered highly informative with allele frequencies p(HWS) ≥0.9, and q(LWS) ≥0.7 or vice versa. All individuals in the AIL (*n *= 1529) were genotyped for these markers using the GoldenGate assay (Illumina, CA) at the SNP technology platform in Uppsala (Sweden).

**Table 2:** Chromosome segments selected for the replication study

Chromosome	QTL segment*	Start (bp)**	End (bp)**
1	*Growth1*	169,634,954	181,087,961
2	*Growth2*	47,929,675	65,460,002
2	*Growth3*	124,333,151	133,581,122
3	*Growth4*	24,029,841	68,029,533
4	*Growth6*	1,354,213	13,511,203
4	*Growth7*	85,459,943	88,832,107
5	*Growth8*	33,696,791	39,052,438
7	*Growth9*	10,916,819	35,491,706
20	*Growth12*	7,109,709	13,899,993

*Jacobsson et al. 2006 [10]; **position as in Chicken genome assembly of May 2006

### IBD estimation

When detecting QTL by the variance component method, the covariance matrix (Π) of the random QTL effect must be estimated as an IBD matrix i.e. a n*n matrix that contains, at a given genomic position, the expected number of alleles IBD between all pairs of individuals in the studied population or pedigree [1]. Therefore, to perform a genome-scan for QTL using a variance component based analysis, an IBD matrix is needed for each tested genome position τ.

IBD matrices can be estimated using methods based on descent tree likelihood [15], by Monte Carlo Marcov Chain methods [16], or deterministically [17]. For the present study, we used a deterministic IBD estimation method that utilizes pedigree, genotype and haplotype information to infer IBD probabilities based on the approach described by Pong-Wong et al. [18]. The use of haplotype information together with deterministic IBD estimation is, in the present case, motivated by its computational efficiency.

Most IBD estimation approaches [15-18] are based on genotype and pedigree information only, whereas marker-phases (or related measurement of the alleles inheritance pathway thorough the pedigree), which are needed to obtain the final IBD probabilities, are estimated alongside the IBD by the algorithm. Deterministic methods [17,18] only use informative markers (where marker phase can be inferred without uncertainty) and therefore only uses part of the available information, whereas iterative methods [15,16] provide better precision in IBD estimation, but also higher computational demand. However, if haplotypes are estimated by an independent routine, and included in the input data, it can increase the amount of information available for the deterministic algorithm. Here, we use an algorithm that first estimates haplotypes using a Genetic Algorithm based method [19], and subsequently includes this haplotype information to improve the accuracy of the deterministic IBD estimation. Because the present study involves analysing several times the same genomic region to test different hypotheses about the population structure, several versions of an IBD matrix are computed for the same locus. This is thus more efficient to isolate the computationally demanding part of the analysis (recursive haplotype estimation) in a preliminary step that only needs do be done once for each genomic region, and then to adopt a fast deterministic IBD estimation algorithm for the second step that is repeated several times for each locus.

Due to the recursive approach, the deterministic IBD estimation algorithm [18] is also flexible. IBD probabilities are computed recursively from the first (F0) to the last (F8) generation, which makes it possible to include a genetic covariance structure among the founder individuals of the pedigree. This covariance can be computed independently based on population history [20], or based on genetic data [21]. The algorithm simply reads the matrix of covariance between founders of the pedigree as input data, and computes the IBD relationship of the last generations as a function of the given founder population structure.

Here, we estimated IBD probabilities recursively in the AIL pedigree as in [18], taking the covariance among the founders into account [21]. Haplotypes were used as input if they were deemed robust [19] but individual marker genotypes were used when haplotype estimates were uncertain. The estimated IBD matrices at each tested location, τ, were then used as input in the variance component (VC) QTL analysis.

### Variance component QTL analysis

#### VC model

The classical VC approach to map QTL assumes that QTL alleles in the founder population are uncorrelated [22,23]. Since the VC approach makes no distinction between the line origin of the alleles, this approach is not suitable to analyse outbred crosses between divergent lines [2,21]. Therefore we used a VC approach [21] that accounts for correlation among QTL alleles within the founder lines.

Consider the variance component model with uncorrelated founder allele effects:

(1) y=Xβ+v+e

where *y *is the vector of individual phenotypes, **β **the vector of fixed effects, **X **the design matrix of fixed effects, **v **a vector of random QTL effects, and **e **the vector of residual effects. The variance of *y *is then given by

$$V(\text{y})=\Pi \sigma_{v}^{2}+\text{I}\sigma_{e}^{2}$$

where Π is the genotype IBD matrix calculated at chromosome position *τ*, $\sigma_{v}^{2}$ is the genetic variance due to the QTL, **I **is the identity matrix, and $\sigma_{e}^{2}$ is the residual variance.

An alternative, and equivalent, presentation of model (1) is [24,21]:

(2) y=Xβ+Zv*+e

where v* is the vector of the effects of *m *independent and identically distributed (iid) base generation QTL alleles, assumed normally distributed with variance $\frac{1}{2}\sigma_{v}^{2}$, $\frac{m}{2}$ is the number of individuals in the base generation, **Z **is an incidence matrix of size *n *× *m *that relates individuals with the QTL alleles in the base generation, and **e **is the residual vector with variance $\sigma_{e}^{2}$.

The assumption of uncorrelated QTL allele effects in the base generation, implies that in model (2): V(v*) = $\frac{1}{2}\sigma_{v}^{2}I_{m}$ where I_m _is an identity matrix of size *m × m*. Equivalently in model (1), the sub-matrix corresponding to the first $\frac{m}{2}$ rows and the first $\frac{m}{2}$ columns of Π (i.e. IBD relationships between the founders at locus *τ *) is an identity matrix.

In crosses between divergent lines, QTL allele effects should be correlated with the origin of the base generation line. This is the underlying assumption of common linear regression based methods used for QTL mapping in outbred crosses [25,26]. To consider the correlation of the alleles in the founder lines when mapping QTL, under the assumption that QTL are not fixed within the founder lines, we introduce *ρ*_*A *_and *ρ*_*B *_as the correlations between the effects of founder QTL alleles from lines A and B, respectively. Then, the covariance structure of v* is not $\frac{1}{2}\sigma_{v}^{2}I_{m}$ any longer as in model (2), but instead:

$$V\left(v^{*}\right)=\frac{1}{2}\sigma_{v}^{2}\left(\begin{array}{cccccccc} 1 & \rho_{A} & & & & & & \\ \rho_{A} & 1 & & & & & & \\ & & 1 & \rho_{B} & \rho_{B} & \rho_{B} & \rho_{B} & \rho_{B}\\ & & \rho_{B} & 1 & \rho_{B} & \rho_{B} & \rho_{B} & \rho_{B}\\ & & \rho_{B} & \rho_{B} & 1 & \rho_{B} & \rho_{B} & \rho_{B}\\ & & \rho_{B} & \rho_{B} & \rho_{B} & 1 & \rho_{B} & \rho_{B}\\ & & \rho_{B} & \rho_{B} & \rho_{B} & \rho_{B} & 1 & \rho_{B}\\ & & \rho_{B} & \rho_{B} & \rho_{B} & \rho_{B} & \rho_{B} & 1 \end{array}\right)$$

where *ρ*_*A *_and *ρ*_*B *_are estimated as in [21]. When QTL alleles are fixed in the founder lines, as assumed in linear regression based QTL mapping [25,26], *ρ*_*A *_= *ρ*_*B *_= 1.

The example given here illustrates a case where the founder population includes one individual from line A and three individuals from line B. The AIL population studied in the present article was obtained by crossing 30 LW with 29 HW animals. Since average body weights can vary considerably between generations (Table 1) and sexes, we fitted a mixed linear model with generation and sex as fixed effects.

#### QTL detection scan

We computed the score statistic at each marker as in [21] to test for QTL in the nine chromosome regions. Markers were assigned to their genomic locations using the dense consensus chicken genetic map [27,28].

First, we computed the score statistic at 1 cM intervals in the genome, using model A:

y=Xβ+v+e

without including a polygenic effect in the model.

Second, we calculated the score statistic at each marker, using model B:

y=Xβ+a+v+e

which differs from model A by including a random polygenic effect (*a*) in the model. The polygenic effects were calculated based upon the additive genetic relationship matrix between all animals in the pedigree.

#### Estimation of QTL allele fixation within lines

When a significant QTL was detected in the chromosome segment scanned with either of the two alternative hypotheses (A or B), we tested whether the level of fixation within lines (*ρ) *was significantly different from zero (i.e. H_0_: *ρ *= 0). For simplicity, the correlation between effects of founder QTL alleles was assumed to be the same in both lines. Therefore, the same value of *ρ *was considered for the LWS and HWS lines. A likelihood ratio test was used to compare three alternative models that were defined based on assuming i) independence of alleles (*ρ *= 0), ii) fixation of alleles (*ρ *= 1), and iii) segregation of alleles (0 <*ρ *< 1). When model iii) was declared most likely (0 <*ρ *< 1), correlations within the LWS and HWS lines (*ρ*_*A *_and *ρ*_*B*_) were estimated separately following the same approach as in the Flexible Intercross Analysis (FIA) described by Rönnegård et al. (2008) [21].

#### Significance thresholds

Significance thresholds for the scans were derived using randomization testing. Residuals estimated from the null model **y **= **Xβ **+ **e **(model A) or **y **= **Xβ **+ *a *+ **e **(model B) were permuted. One thousand replicates of the phenotypic data were simulated with $\text{y}=\text{X}\tilde{\beta }+\overset{⌣}{\varepsilon }$, (or $\text{y}=\text{X}\tilde{\beta }+ã+\overset{⌣}{\varepsilon }$ for model B) where $\overset{⌣}{\varepsilon }$ is the vector of permuted residuals, $\tilde{\beta }$ and ã are the estimated fixed and random effects obtained from the null model. For each replicate, the score statistic was calculated at every tested position in the selected region. As in Valdar et al. [29], the maximum score value from each replicate was then fitted to a generalized extreme value distribution using the evd library in R [30,31]. 5% and 1% significance thresholds were then estimated respectively by the 95% and 99% quantiles of the fitted distribution.

## Results

### QTL detection scan

**Figure 1:**
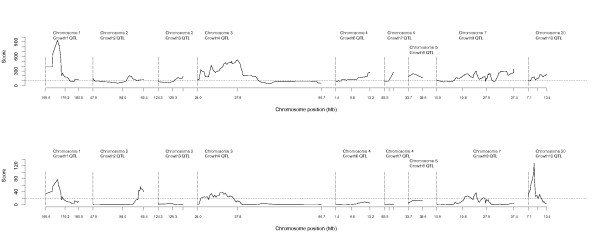
**QTL scan in a nine generation AIL pedigree with model A (A) and model B (B)**. The score statistic is plotted against the position in Mb for each of the nine analyzed chromosome segments; the 5% experiment-wise significance threshold is given as a horizontal dashed line

In scans using model A (without a polygenic effect), all nine QTL regions identified with the original F_2 _population [10,11] also contained significant QTL with the AIL pedigree. A QTL profile including all chromosome segments is in Figure 1A. The QTL on chromosome 7 (*Growth9*) was split into multiple peaks that together covered most of the selected chromosome segment, whereas the other segments *Growth1*, *Growth2*, *Growth3*, *Growth4*, *Growth6*, *Growth7*, *Growth8 *and *Growth12 *contained a single QTL peak. In the scans using model B (with a random polygenic effect), only *Growth1, Growth2*, *Growth4, Growth9 *and *Growth12 *contained significant QTL (Figure 1B).

Estimates of locations and genetic effects for all the QTL are summarized in Table 3. QTL positions reported in Table 3 are the maximum point (mode) of the curve. As model B provided the strongest statistical support for the QTL, the allele effects reported in Table 3 were measured at the location supported by model B when the peaks for both models did not coincide.

**Table 3:** Genomic location and genetic effect of the replicated QTL

QTL	Chromos	Position (bp)* Model A	Position (bp)* Model B	Average allele effect LWS alleles	Average allele effect HWS alleles
*Growth1*	1	173,709,609	173,709,609	-30.4 ± 6.7	36.4 ± 6.5
*Growth2*	2	60,710,066	64,098,899	-0.32 ± 7.3	13.9 ± 7.2
*Growth3*	2	130,113,120	NA	22.4 ± 6.6	-14.6 ± 6.8
*Growth4*	3	37,345,273	33,882,778	-7.2 ± 6.5	16.8 ± 6.7
*Growth6*	4	12,635,648***	NA	-11.2 ± 10.3	44.0 ± 12.4
*Growth7*	4	87,824,674***	NA	2.7 ± 7.129	-4.5 ± 7.0
*Growth8*	5	35,422,843***	NA	-16.6 ± 6.9	20.8 ± 6.9
*Growth9.1*	7	21,585,687	21,585,687	-15.2 ± 6.6	16.6 ± 6.7
*Growth9.2*	7	32,701,577***	NA	-5.4 ± 6.7	11.5 ± 6.7
*Growth12*	20	11,137,932	9,100,162	-11.5 ± 6.4	19.8 ± 6.2

***position as in Chicken genome assembly of May 2006

*Growth1 *and *Growth9.1 *mapped to the same position in scans with model A or B. *Growth4 *was located within the same large interval (24 to 38 Mb) as previously, with a 4 Mb difference between estimates from scans using models A and B. Similarly, for *Growth2 *the same location was found with a 4 Mb difference between estimates from scans using models A (60 Mb) and B (64 Mb). For *Growth12*, two peaks were detected at 9 and 11 Mb with both models but the peak at 9 Mb was more significant in the scan with model B. Average effects of QTL alleles at *Growth1*, *Growth2*, *Growth4*, *Growth6*, *Growth8*, *Growth9 *and *Growth12 *were as expected: negative for the LWS alleles and positive for the HWS alleles. Effects of QTL alleles at *Growth3 *and *Growth7 *were cryptic, contrary to the original observation [10], with a positive effect for LWS alleles and a negative effect for HWS alleles.

### QTL fine-mapping

Since QTL-mapping with AIL increases resolution compared to F_2 _designs, it is possible to test whether the studied segments contain one or several regions that contribute to the QTL effect. The QTL profiles initially obtained (Figure 1) suggested that several segments might contain more than a single signal. Therefore, a second scan was performed for the segments for which the detection of a QTL was replicated with model A. In this case, only the phenotypes of individuals from the last generation (F_8_, n = 400), with the lowest linkage disequilibrium, were included. IBD between these individuals were, however, computed using the genotypes from all individuals in the pedigree to obtain the best possible QTL genotype estimates. In this scan, a two-QTL model was fitted to evaluate the evidence for multiple linked QTL in these regions. In these analyses, the genetic variance of one of the two QTL in the region was included in the null model of the VC analysis while that of the other was included as a main effect. These analyses showed that there were two independent effects in the *Growth9 *region at approximately 20 Mb and 35 Mb and these were named *Growth9.1 *and *Growth9.2*, respectively. The two regions *Growth9.1 *and *Growth9.2 *are considered as different QTL in the rest of the manuscript (Table 3).

**Figure 2:**
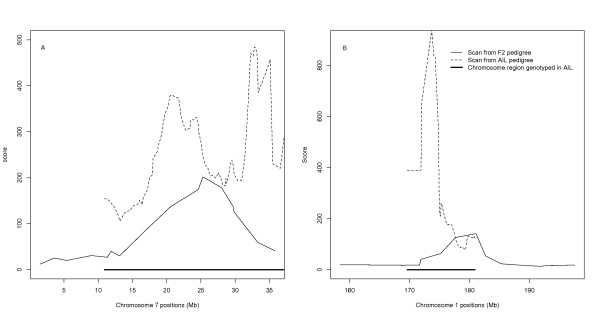
Comparison of the QTL profiles for *Growth9 *(A) and *Growth1 *(B) in the original F_2 _pedigree and the nine generation AIL pedigree

For several QTL segments, the peak obtained with the AIL was narrower than with the original F2 population, which illustrates the higher resolution of QTL mapping using AIL. Figure 2 compares QTL peaks obtained with the F_2 _population and with the AIL for *Growth1 *on chromosome 1 and *Growth9 *on chromosome 7. The peak width of *Growth1 *with the AIL was about 1/3 of the peak with the F_2 _design (Figure 2B). The single QTL (*Growth9*) on chromosome 7 identified with the F_2 _pedigree could be separated into two narrow QTL with the AIL (Figure 2A). Due to recombinations accumulated in successive generations, the size of the QTL region was also considerably smaller in the scan carried out with the AIL than that with the F_2 _design for *Growth2*, *Growth4 and Growth12 *QTL, whereas the peaks for *Growth3, Growth6, Growth7 and Growth8 *still covered the entire genotyped segment (Figure 1).

### Estimation of allele correlation within lines

A preliminary analysis indicated that independence of the alleles was common in the present pedigree. We hereafter considered allele independence as the null hypothesis and then tested for possible fixation or segregation within the lines. When comparing the likelihood of these two alternative hypotheses of fixation (ρ = 1) or segregation (0 < ρ <1) of QTL alleles within founder lines, segregation was identified for *Growth1 *(P < 0.02) and for one (*Growth9.1*) of the two QTL on chromosome 7 (P < 0.05). For the other QTL, the model assuming independence (ρ = 0) of the alleles could not be rejected.

At *Growth1*, the estimated correlation of the allelic effects was 0.14 in the LWS line and 0.74 in the HWS line. For *Growth9.1*, the within-line correlation was 0.61 for LWS and 0.88 for HWS. For these two regions, the FIA model [21] indicates a higher level of fixation within the HWS line than within the LWS line.

**Figure 3:**
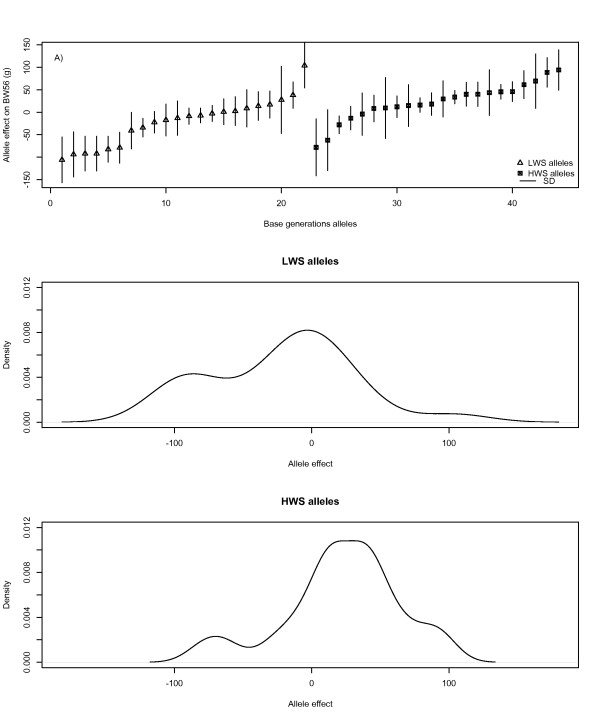
**Estimated allele effects on bodyweight at 56 days of age for the base generation alleles of the *Growth1 *QTL in the AIL pedigree**. In A, allelic effects are plotted sorted by effect-size and line origin, in B and C density distributions of the allele substitution effect are given for LWS (B) and HWS (C) alleles, respectively

For each base generation allele at *Growth1 *that was transmitted to at least seven descendants, the substitution effect was calculated (see Figure 3A). Alleles from both HWS and LWS lines had both positive and negative effects on body weight, with more dispersion of the effects in the LWS line (Figures 3B and 3C), where allelic effects varied from-105 g to +103 g (mean = -21 g). Alleles from the HWS line had mostly positive effects, ranging from -75 g to +90 g (mean = 22 g)

## Discussion

Analysis of data obtained with an advanced intercross line originating from inbred founders is straightforward because alternative alleles of the markers are fixed in each founder line. In such designs, it is sufficient to collect the data from later generations in the pedigree and then use standard QTL mapping software developed for inbred intercross populations for the analysis. The major difference between the F_2 _and the following generations of the AIL is the increase in recombination events. However, QTL analysis with an AIL originating from outbred founders is not trivial because fixation of neither markers nor QTL can be assumed in the original lines. In order to maximize the power to replicate QTL detection and fine-mapping using an AIL produced from outbred founders, we propose that the genotypes and phenotypes of all the individuals in the pedigree and not just of those from later generations should be collected and analysed. In our work, we have applied this strategy to an experimental chicken dataset and analyzed the data for nine genomic regions for which significant or suggestive QTL had been previously identified with an F_2 _design between the same chicken lines [10,11]. Two alternative models were used for QTL detection: (1) a model (A) without a random polygenic effect, which detected significant QTL in all nine regions and (2) a more stringent model (B) that included a random polygenic effect, which reduced the number of significant QTL to five regions. This difference in number of QTL detected is due to the fact that the covariance matrix of the polygenic effect included in model B is by definition very similar to the covariance matrix of the QTL effect when marker information is poor. The information content estimated from the IBD coefficients [32] appeared indeed to be lower in some regions where QTL was detected in model A but not in model B (*Growth 3 Growth 7 Growth 8*). However, one of the low information content regions (*Growth 9.1*) was nevertheless detected in both models. This makes it difficult to determine whether the loss of QTL with model B is due to false positive signals obtained with model A, or to the fact that marker information content is simply too low to distinguish between a QTL effect and a polygenic effect in a multigenerational intercross pedigree. Based on our results, it can be concluded that *Growth1, Growth2, Growth4, Growth9.1 *and *Growth12 *contain QTL that are strongly supported by both models. The allelic effects in these regions are positive for the HWS allele and negative for the LWS allele, as expected in an AIL resulting from a cross between two divergently selected lines. In addition to these five significant QTL regions detected with both models, the remaining regions (*Growth3, Growth6, Growth7, Growth8*, and *Growth 9.2*) are likely to contain QTL based on the analyses using model A. This may be resolved by further analyses with more informative markers.

Eight QTL acted in the same direction in both the F_2 _population and the AIL i.e. the effect of their HWS alleles were additive and led to higher BW56 as in [10], while two QTL acted in opposite directions. This difference can be explained by the lack of fixation of QTL alleles within the founder lines (as illustrated by the range of estimated allelic effects in Figure 3), where alleles with positive and negative effects were present in both the HWS and LWS lines. Since multiple alleles exist in both lines, the estimated difference between the average effects of alleles inherited from HWS and LWS animals is a mixture of high and low effect alleles. Thus, when analyzing a particular generation in a population, the results will depend on the actual set of alleles sampled from a limited number of ancestors. As the number of founders for each generation is rather small, genetic drift will have an influence on the results. It is worth noting that several of the QTL confirmed in our study were not detectable using methods that assume allelic fixation in the founder lines. Their detection relied on the use of a variance component approach that does not assume fixation. Another potential explanation for the difference in observed effects is epistasis, which is known to be strong among QTL in this pedigree [33]. Therefore, the size of the genomic region containing the QTL and the direction of its effect depend on the genetic background at other loci. Differences in allele frequencies at interacting loci might influence the marginal effects of QTL and even lead to genetic effects that change direction depending on the allele frequency at the loci with which it interacts. Although an in-depth study of epistasis is beyond the scope of this paper, preliminary tests provide some strong evidence for epistasis in this pedigree, with, e.g., the LWS allele of *Growth3 *having a positive effect when combined with the HWS allele of *Growth12 *but a negative effect when combined with the LWS allele. A third possibility is that recombination in subsequent generations has disrupted linkage disequilibrium between linked QTL so that the QTL effect at the position tested in the current study deviates significantly from the one observed in the F_2 _generation.

The nine selected chromosome segments were first scanned for single QTL using a variance component approach. The assumption of a single QTL in each segment appears valid for all regions but *Growth9*. When including all phenotype data from the F_2_-F_8 _generations, the segment containing *Growth9 *had a complex QTL profile indicating multiple independent genetic effects. A two-QTL analysis was then performed including only the phenotypic data from the F_8 _generation. Since linkage disequilibrium is lower in this last generation, resolution should be higher when using this smaller dataset. In this analysis, the QTL region splits into two significant QTL at 20 and 35 Mb (Figure 2A). The peak observed between the two peaks at 30 Mb in the single-QTL scan is not significant, indicating that it is most likely a false "ghost" signal due to linkage with the two neighbouring regions.

Our scan permits the detection of QTL that segregate within the parental lines. Thus, it is a powerful approach to detect QTL in crosses produced from divergent outbred lines. It identified ten QTL in nine distinct chromosome regions. Two regions (*Growth1 *and *Growth9.1*) showed significant (p < 0.05) within-line correlations between allele effects. The estimated correlation of the within-line allele effects, calculated from the FIA model, was higher within the HWS line (0.74) then within the LWS line (0.14) for *Growth1*. This was consistent with the variability among allele substitution effects shown in Figure 3, which is larger among LWS alleles than among HWS alleles. For the remaining eight QTL, we could not reject the null-hypothesis of independent allelic effects (even within-line). Since the parental lines had been divergently selected and display highly divergent phenotypes, a stronger correlation of allelic effects in the founder lines was expected. However, segregation within lines is not unlikely, because the lines were not inbred, the QTL effects were rather small and the time of divergence between the lines was relatively short. This finding could, however, also be explained by a lack of power in the segregation analyses in this pedigree since it contains a large number of founder alleles with rather few observations for each inherited allele.

## Conclusions

Here, we have produced, genotyped and analyzed a large AIL obtained from outbred parents. Most of the QTL originally detected in the F_2 _population were confirmed, which indicates that appropriately sized replication populations and powerful statistical tools are crucial to refine original QTL findings and dissect the genetics underlying complex phenotypes. Replicating the detection of the QTL and fine-mapping their location with an AIL strengthen the original findings, and validate AIL as a valuable tool to explore the genetic basis of complex traits. We also believe that the methods available now to analyze outbred intercross populations can be useful for in-depth genetic studies of a wider range of organisms and can provide answers to research questions that are not approachable using inbred model organisms.

## Competing interests

The authors declare that they have no competing interests.

## Authors' contributions

FB analyzed the data, OC, PBS and LA designed the experiment, PBS was responsible for animal experiments, PBS and PW performed the phenotyping, WE, PW and OC were responsible for marker selection and genotyping, FB, WE, OC and LR designed and contributed to the statistical analysis. FB and OC wrote the first draft of the manuscript and all co-authors contributed to the final version.
